# Prevalence and Prognostic Significance of Sarcopenia in Gynecologic Oncology: A Systematic Review and Meta‐Analysis

**DOI:** 10.1002/jcsm.13699

**Published:** 2025-02-02

**Authors:** Chen Jiang, Qin Chen, Danping Yu, Qianqian Zhou, Cong Tang, Chenping Qiao

**Affiliations:** ^1^ Department of Gynecology Women’s Hospital of Nanjing Medical University, Nanjing Women and Children’s Healthcare Hospital Nanjing Jiangsu China

**Keywords:** gynaecologic oncology, meta‐analysis, prevalence, prognosis, sarcopenia

## Abstract

**Background:**

Sarcopenia in gynaecologic oncology patients has garnered increasing attention, but its prevalence has not been comprehensively summarized. This study aims to integrate the prevalence of sarcopenia in this population through systematic evaluation and meta‐analysis, providing a reference for future clinical research.

**Methods:**

A computerized search of PubMed, Embase, The Cochrane Library, Web of Science and other databases was conducted to collect relevant literature on the prevalence of sarcopenia in gynaecologic oncology patients and its impact on prognosis, with a timeframe from the inception of the databases to July 2024. Two researchers independently screened the literature, extracted information and assessed the risk of bias. After evaluating the risk of bias, a meta‐analysis was performed using RevMan5.4 software. This systematic review was conducted using a previously published study protocol (PROSPERO: CRD42024565094).

**Results:**

A total of 24 studies encompassing 4136 patients were included. The meta‐analysis revealed that the prevalence of sarcopenia in gynaecologic oncology patients was 38.8% (*I* ^2^ = 96%, 95% CI [0.49–0.79], *p* < 0.001). Subgroup analysis indicated that the prevalence of sarcopenia was higher among patients with endometrial cancer or ovarian cancer, those over 60 years of age, individuals with a body mass index (BMI) greater than 25 kg/m^2^, those diagnosed using the psoas muscle index (PMI) and patients assessed at the L4 vertebra. Overall survival (OS) was significantly lower in patients with gynaecologic tumours combined with sarcopenia compared to those with gynaecologic tumours alone. However, no significant differences were observed in progression‐free survival (PFS), mortality or length of hospital stay.

**Conclusion:**

Sarcopenia has a high prevalence in gynaecologic oncology patients. Healthcare professionals should prioritize early screening and preventive measures for high‐risk patients.

## 1 Introduction

Sarcopenia, first introduced by Rosenberg in 1989, was initially defined as a pathological condition characterized by the age‐related loss of skeletal muscle mass and strength. This decline is associated with a reduction in physical mobility and an increased risk of adverse outcomes, including falls and disability [1]. In 2018, the European Working Group on Sarcopenia (EWGSOP) revised the definition of sarcopenia, characterizing it as a progressive and generalized skeletal muscle disorder associated with an elevated risk of falls, fractures, physical disability, mortality and other adverse outcomes. Patients with tumours experience heightened catabolic activity and reduced anabolic processes, leading to a higher prevalence of sarcopenia compared to the general population. When linked to malignancies, this condition is called tumour‐associated sarcopenia [2]. The Asian Working Group for Sarcopenia (AWGS) for sarcopenia prioritizes the practicality of the diagnostic process compared to the EWGSOP criteria, with diagnostic thresholds adapted to the physical characteristics of Asian populations. Although both criteria align in their focus on decreased muscle mass and reduced physical function, they differ in the specific screening tools, diagnostic procedures and thresholds employed [3]. A systematic review of 35 studies involving 6894 patients found that the prevalence of pre‐treatment sarcopenia in cancer patients was 38.6% [4]. There is a recognized association between tumour‐associated sarcopenia and tumour cachexia, though Aust et al. [5] have noted distinct differences in their pathogenesis. Sarcopenia primarily results from age‐related endocrine changes and insufficient protein intake. In contrast, inflammation and oxidative stress drive muscle atrophy in cachexia, leading to muscle and adipose tissue loss. Recent studies suggest that muscle loss may also occur in tumour patients who have not yet reached a malignant state. Unlike cachexia, patients with tumour‐associated sarcopenia may not experience anorexia and may exhibit both skeletal muscle loss and fat accumulation [6, 7].

The diagnosis of sarcopenia typically involves assessing low muscle mass, muscle strength and performance, often using imaging modalities such as computed tomography (CT), magnetic resonance imaging (MRI) or dual‐energy x‐ray absorptiometry (DEXA) [8, 9]. CT imaging is the most used method for measuring skeletal muscle at the third lumbar spine area and has been shown to correlate well with whole‐body muscle mass [10, 11]. The prevalence of sarcopenia among cancer patients varies significantly, mainly due to the lack of standardized data. This variation is primarily attributed to differences in the definitions of sarcopenia and the diverse methods used to assess it. A Mendelian randomization study by Huang et al. [12] indicated that sarcopenia may be linked to poor surgical outcomes and an increased risk of mortality in patients with ovarian cancer. This finding provides genetic evidence supporting a causal association between sarcopenia and ovarian cancer, highlighting the necessity for early prevention and intervention strategies to identify modifiable risk factors at an early stage and ultimately reduce the risk of adverse outcomes. At the same time, malnutrition has been shown to be a major problem in gynaecologic cancer patients, especially those with ovarian cancer where there is an established association between obesity and the risk of endometrial and ovarian malignancies. Due to the absence of precise data on the prevalence of sarcopenia in patients with gynaecologic cancers, this study conducted a meta‐analysis to assess its prevalence and its impact on prognosis. The findings aim to provide a foundation to support future research in this area.

## 2 Methods

This systematic review was conducted using a previously published study protocol (PROSPERO: CRD42024565094). During the synthesis of this review, the Enhancing Transparency in Reporting the Synthesis of Qualitative Research (ENTREQ) statements were utilized as guidelines [13].

### 2.1 Literature Search Strategy

Computerized PubMed, Embase, The Cochrane Library and Web of Science searches were conducted using subject terms and free words. The search terms included ‘sarcopenia/skeletal muscle*/skeletal muscle*/muscle mass/muscle*’ and ‘ovarian/endometrial neoplasms/uterine neoplasms/uterine cervical neoplasms/genital neoplasms, female/vaginal neoplasms/vulvar neoplasms/choriocarcinoma, non‐gestational/choriocarcinoma’. Additionally, references in the incorporated literature were hand‐searched as a supplement. Using PubMed as an example, the search formula is shown in Table S1. The search covered the period from establishing each database to July 2024.

### 2.2 Literature Inclusion and Exclusion Criteria

Inclusion criteria are as follows: (1) Study types were cross‐sectional, case–control and cohort studies. (2) Study subjects were patients with gynaecologic oncology, including ovarian, endometrial, cervical, vaginal, vulvar, and fallopian tube cancers, who were ≥ 18 years of age. (3) The study provided a precise diagnosis of sarcopenia. (4) The literature described the prevalence of sarcopenia in patients with gynaecologic oncology, its prevalence and its prognostic impact or provided relevant data on the prevalence of sarcopenia in gynaecologic oncology. Exclusion criteria are animal studies, conference papers and duplicate publications.

### 2.3 Literature Screening and Data Extraction

Two researchers independently conducted literature screening, information extraction and cross‐checking. In cases of disagreement, a third party was consulted to assist in the judgement. Authors were contacted as needed to obtain additional information when it was lacking. The screening process involved an initial review of titles and abstracts, followed by a full‐text review of potentially relevant studies.

### 2.4 Literature Quality Assessment

The risk of bias in cross‐sectional studies was assessed using 11 evaluation criteria recommended by the Agency for Healthcare Research and Quality [14]. The criteria included (1) clear identification of the information source; (2) listing of inclusion and exclusion criteria for both exposed and non‐exposed groups; (3) specification of the period for patient identification; (4) description of whether subjects were consecutive or from a population source; (5) consideration of whether subjective factors of the evaluator influenced the study population; (6) documentation of quality assurance measures; (7) explanation of reasons for excluding patients; (8) description of measures to evaluate and/or control for confounders; (9) explanation of how missing data were handled in the analyses, if applicable; (10) summary of the response rate and completeness of data collection; and (11) if follow‐up was available, description of the percentage of patients with incomplete data or follow‐up results. Each criterion was scored as ‘yes’ (1 point), ‘no’ (2 points) or ‘not clear’ (0 points), resulting in a total score out of a possible 11 points. The quality was categorized as follows: 0–3 points indicated low quality, 4–7 points indicated moderate quality, and 8–11 points indicated high quality. The risk of bias in cohort studies was assessed using the Newcastle–Ottawa Scale [15], which comprises three dimensions with a total of eight criteria: selection of study subjects (4 criteria), comparability between groups (2 criteria) and outcome measures (3 criteria). Each study was scored out of a possible 9 points. A score of 0–4 indicated low quality, 5–6 indicated moderate quality, and 7 or higher indicated high quality. The quality of the literature was evaluated independently by two researchers, with a third party consulted to resolve any discrepancies between the researchers' scores.

### 2.5 Statistical Analyses

Meta‐analysis was conducted using RevMan5.4 software. Heterogeneity among studies was assessed using the *Q* test (*p* value) and the *I* ^2^ statistic; a fixed‐effects model was applied if there was no significant statistical heterogeneity (*I* ^2^ ≤ 50% and *p* > 0.10), whereas a random‐effects model was used otherwise. Two researchers evaluated clinical heterogeneity based on their expertise and clinical experience to exclude significant variability in clinical characteristics. Methodological heterogeneity was assessed by examining differences in study designs and results. Sources of heterogeneity were explored through subgroup analyses based on disease diagnosis, age, body mass index (BMI), diagnostic criteria and study location. If no heterogeneity was detected within each subgroup or if the meta‐regression coefficient had a *p* value < 0.05, the factor was considered a potential source of heterogeneity. The prognostic impact of sarcopenia in gynaecologic oncology patients was analysed descriptively due to incomplete and inconsistent data types. Publication bias was assessed using Egger's test with a significance level of *α* = 0.05, and the stability of the results was tested through a sensitivity analysis, which involved sequentially excluding each study.

## 3 Results

### 3.1 Literature Search Results

**FIGURE 1:**
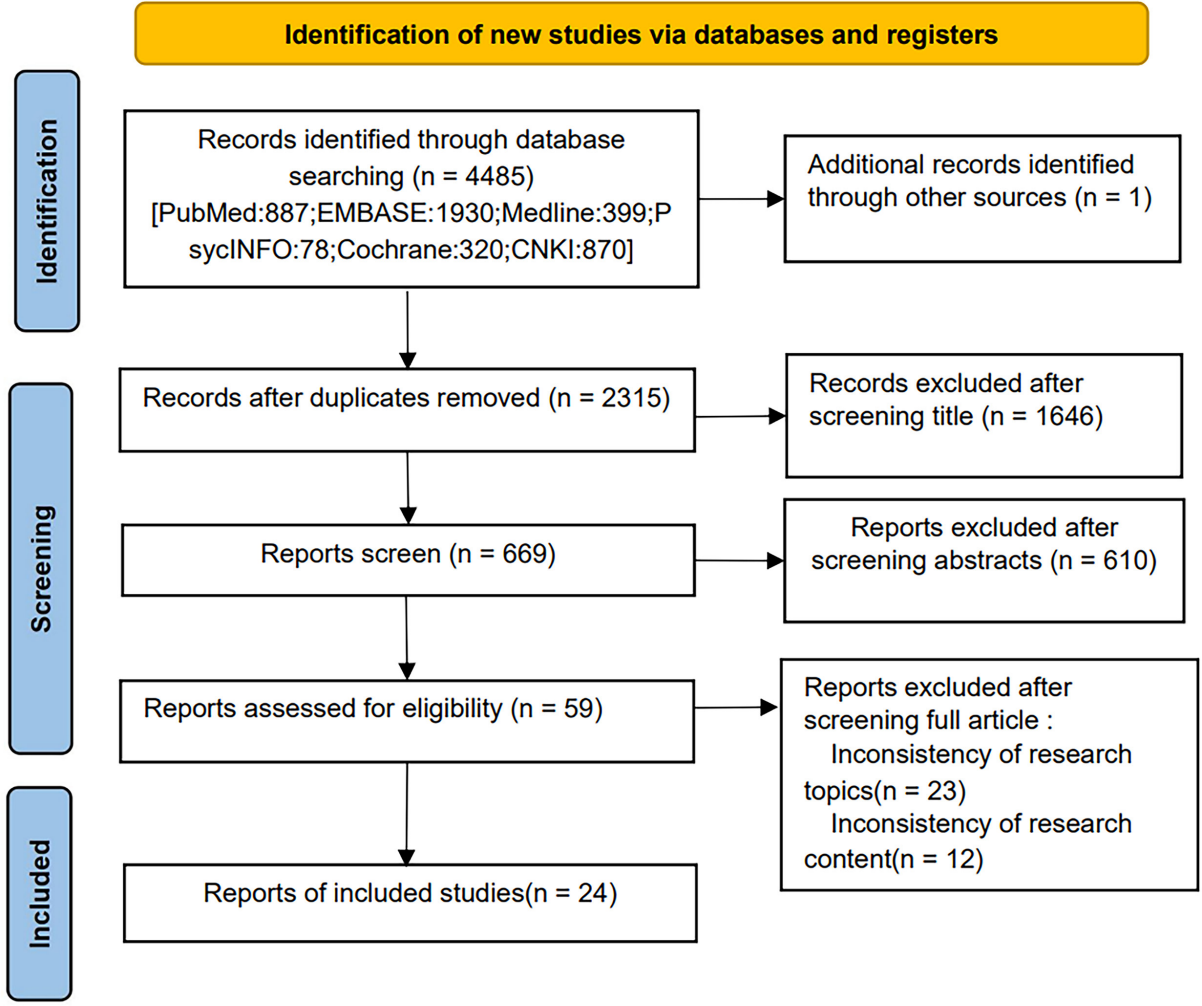
Flow chart of literature selection.

A total of 4485 articles were identified during the initial screening. After a detailed, tiered screening process, 24 studies [16, 17, 18, 19, 20, 21, 22, 23, 24, 25, 26, 27, 28, 29, 30, 31, 32, 33, 34, 35, 36, 37, 38, 39] were included, encompassing a total of 4352 patients with gynaecological tumours, of whom 1600 were diagnosed with sarcopenia. The flow chart of the literature selection is illustrated in Figure 1.

### 3.2 The Quality Assessment of the Included Literature

The 24 studies comprised one cross‐sectional study and 23 cohort studies. The basic characteristics of these studies are summarized in Table 1. The cross‐sectional study received a literature quality score of 8, with ‘unclear’ results for Entry 5 (‘whether the evaluator's subjective factors overshadowed the rest of the study population’) and ‘no’ for Entry 9 (‘If possible, explain how missing data were handled in the analysis’). The cohort studies had quality scores ranging from 6 to 8, indicating medium to high quality. The detailed literature quality scores are provided in Table S2.

**TABLE 1:** Characteristics of the included studies (*n* = 24).

Author (year)	Country	Type	Research objectives	Survey instrument	Age(years)/mean (SD) or range	Sample size	Timepoint	Rate (%)	BMI	Measured value	Outcome	Define
Yamada et al. (2022) [22]	Japan	a	Gynaecological cancer	DEXA	60 (29–89)	475	Before treatment	45 (9.5)	23.9 ± 4.94	6.02 (4.10–10.03)	—	SMMI < 5.4 kg/m^2^ and handgrip strength < 18.0 kg or SMMI < 5.4 kg/m^2^ and low physical capability (SPPB ≤ 9 or Conduct standing test time > 12 s)
Aust et al. (2015) [5]	Australia	b	Ovarian cancer	CT (L3)	60 ± 13	140	Before treatment	39 (28.9)	24.9 ± 4.8	44.9 ± 7.4	①②	SMI < 41.0 cm^2^/m^2^
Kuroki et al. (2015) [14]	USA	b	Endometrial cancer	CT (L3)	65.9 ± 10.4	122	Before treatment	61 (50)	35.3 ± 10.2	1.7 ± 0.5	③	PMI < 4.33 cm^2^
Kumar et al. (2016) [13]	US	b	Ovarian cancer	CT (L3)	64.6 ± 10.6	296	Before treatment	132 (44.6)	—	39.9 ± 6.6	①②	SMI < 39.0 cm^2^/m^2^
Bronger et al. (2016) [6]	Isar	b	Ovarian cancer	CT (L3)	65 (33–85)	128	At diagnosis	16 (12)	25.0 ± 5.4	—	①②	SMI: 38.5cm^2^/m^2^
Rutten et al. (2016) [16]	The Netherlands	b	Ovarian cancer	CT (L3)	66.5 ± 0.8	123	Before treatment	62 (50.4)	25.9 ± 0.5	41.7 ± 0.5	①	SMI:41.5 cm^2^/m^2^ (median in the study)
Silva de Paula et al. (2017) [27]	Brazil	b	Gynaecological cancer	CT (L3)	—	250	Before treatment	56 (22.4)	—	43.4 (27.4–66.3)	—	SMI ≤ 38.9 cm^2^/m^2^
Conrad et al. (2017) [8]	USA	b	Ovarian cancer	CT (L4)	55 ± 11	284	Before treatment	153 (54)	28.0 ± 6.9	2.80 ± 0.80	③④	PMI < 2.8 cm^2^/m^2^
Yoshikawa et al. (2017) [24]	Japan	b	Ovarian cancer	CT (L5)	61 (33–78)	76	Before treatment	38 (50)	—	58.3 (32.6–99.9)	—	PMI: No define
Ataseven et al. (2018) [4]	Germany	b	Ovarian cancer	CT (L3)	60 (21–89)	323	Before treatment	152 (47.1)	—	41.5 ± 6.3	①	SMI < 41.0 cm^2^/m^2^
Nattenmüller et al. (2018) [15]	Germany	b	Gynaecological cancer	CT (L3–L4)	62.88 ± 13.53	189	At diagnosis	66 (34.8)	26.81 ± 6.31	43.1 ± 24.3	—	SMI: No define
Sánchez et al. (2019) [17]	Mexico	b	Cervical cancer	CT (L3)	50.45 ± 11.4	55	Before treatment	18 (33)	25 (16–34)	37.9 (18.3–52.6)	—	SMI < 38 cm^2^/m^2^
Yamada et al. (2021) [21]	Japan	b	Gynaecological cancer	DEXA	53 (34–89)	82	Before treatment	23 (28)	22 (13–41.5)	5.96 (3.56–10.0)	—	SMMI < 5.4 kg/m^2^
Huang et al. (2020) [12]	Taiwan	b	Ovarian cancer	CT (L3)	54.4 ± 10.3	139	Before treatment	48 (34.5)	22.3 ± 3.4	41.9 ± 7.0	①②	SMI < 39.2 cm^2^/m^2^
Fadadu et al. (2020) [10]	USA	b	Ovarian cancer	CT (L3)	64.0 ± 11.2	285	Before treatment	93 (32.6)	27.9 ± 5.7	42.7 ± 6.8	—	SMI < 39.0 cm^2^/m^2^
Donkers et al. (2020) [9]	UK	b	Endometrial cancer	CT (L3)	70 ± 58	176	Before treatment/at diagnosis	61 (35)	29.4 ± 39.4	—	①	SMI < 41 cm^2^/m^2^
Staley et al. (2020) [18]	USA	b	Ovarian cancer	CT (L3)	63.6 (53.3–69.8)	201	Before treatment	119 (59)	26.9 (23.6–32.5)	39.1 (33.5 ~ 45.4)	①②	SMI < 41 cm^2^/m^2^
Yoshikawa et al. (2020) [23]	Japan	b	Cervical cancer	CT (L3)	56.9 ± 13.4	40	Before treatment	23 (57.5)	20.2 ± 3.2	—	①	PMI ≤ 3.72 cm^2^/m^2^
Flores‐Cisneros et al. (2020) [11]	Mexico	b	Cervical cancer	DEXA	49 (39–61)	155	Before treatment	10 (6.5)	28.6 ± 7.4	Handgrip strength: 20.0 ± 4.7	—	Handgrip strength < 20 kg and SMMI < 6.42 kg/m^2^
Yoshino et al. (2020) [25]	Japan	b	Ovarian cancer	CT (L3)	63.5 (43–81)	75	Before treatment	36 (60)	22.7 (16.2–29.5)	—	①	SMI < 39.0 cm^2^/m^2^
Ubachs et al. (2020) [19]	The Netherlands	b	Ovarian cancer	CT (L4)	60.9 ± 8.1	212	At diagnosis	133 (62.7)	20.5 ± 3.5	39.5 ± 5.4	①	SMI: a percentage of change per 100 days decrease of > 2% is considered sarcopenia
Chae et al. (2021) [7]	Korea	b	Ovarian Cancer	CT (L3)	52 (18–83)	82	Before treatment	17 (20.7)	22.6 (16.3–34.1)	—	①③④	SMI < 38.7 cm^2^/m^2^
van der Zanden et al. (2021) [26]	The Netherlands	b	Ovarian cancer	CT (L3)	75.9 (70–89)	213	Before treatment	123 (58)	—	—	③	SMI < 38.5 cm^2^/m^2^
Ubachs et al. (2022) [20]	The Netherlands	b	Ovarian cancer	CT (L3)	65 (22–81)	15	Before treatment	6 (40)	25.8 ± 3.1	41.1 ± 4.8	—	SMI < 39.1 cm^2^/m^2^

*Note:* a, cross‐sectional study; b, cohort study. ① Overall survival refers to the time from the patient's entry into the study to the final death (including the time of death from any cause); ② progression‐free survival refers to the time from the patient's entry into the study to the time of the first metastasis or recurrence of the tumour, or the time of death from any cause; ③ mortality; ④ length of stay.

Abbreviations: OS, overall survival; PFS, progression‐free survival; PMI, the psoas muscle area (cm^2^)/height (m^2^); SMI, skeletal muscle index = muscle area (cm^2^)/height (m^2^); SMMI, skeletal muscle mass index = sum of skeletal muscle content of limbs (kg)/high (m^2^).

### 3.3 Overall Prevalence and Subgroup Analysis of Sarcopenia in Gynaecologic Oncology Patients

**FIGURE 2:**
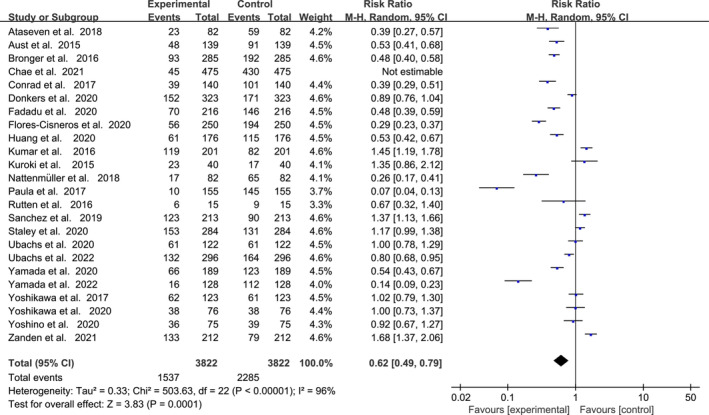
Meta‐analysis results of overall prevalence.

The overall prevalence of sarcopenia among gynaecologic oncology patients ranged from 9.5% to 62.7% across the 24 included studies. Analysis using a random‐effects model revealed that the pooled prevalence of sarcopenia in this patient population was 38.8% (*I* ^2^ = 96%, 95% CI [0.49–0.79], *p* < 0.001), as depicted in Figure 2. Subgroup analyses by disease diagnosis, age, BMI, diagnostic criteria for sarcopenia and CT evaluation site showed varied results. Specifically, the prevalence of sarcopenia was 23.68% (*I* ^*2*^ = 99%, 95% CI [0.35–0.79], *p* = 0.002) in patients with gynaecologic tumours overall, 43.63% (*I* ^2^ = 99%, 95% CI [0.74–1.00], *p* = 0.04) in ovarian cancer patients, 42.5% (*I* ^2^ = 94%, 95% CI [0.66–1.15], *p* < 0.32) in endometrial cancer patients and 32.23% (*I* ^2^ = 100%, 95% CI [0.32–1.30], *p* = 0.22) in cervical cancer patients. Significant differences were observed among subgroups based on age, diagnostic criteria for sarcopenia and evaluation location. Detailed values are provided in Table 2.

**TABLE 2:** Subgroup analysis of the prevalence of sarcopenia in gynaecologic oncology patients.

		Heterogeneity test		Meta‐analysis related results		
Subgroups	Number of included studies	*I* ^2^ (%)		*p*	Prevalence rate (%)	Prevalence (95% CI)
Population						
Gynaecological cancer	3	99	0.002	23.71	0.53 (0.35–0.79)	
Ovarian cancer	15	99	< 0.001	42.12	0.86 (0.74–1.00)	
Endometrial cancer	2	94	0.32	42.33	0.87 (0.66–1.15)	
Cervical cancer	2	100	0.22	32.23	0.64 (0.32–1.30)	
Age (years)						
≤ 60	10	100	0.01	37.08	0.69 (0.52–0.92)	
> 60	11	94	0.16	42.08	0.94 (0.87–1.02)	
BMI (kg/m^2^)						
≤ 25	10	100	0.02	35.7	0.78 (0.63–0.97)	
> 25	5	87	0.006	37.46	0.86 (0.78–0.96)	
Diagnostic criteria						
SMI	15	99	< 0.001	38.62	0.79 (0.69–0.90)	
SMMI	3	100	0.43	14.66	0.71 (0.30–1.68)	
PMI	3	91	0.36	52.5	1.05 (0.95–1.16)	
Position						
CT L3	18	98	0.01	38.53	0.41 (0.20–0.84)	
CT L4	3	95	0.48	50.51	0.22 (0.05–1.06)	
DEXA	3	95	< 0.001	14.66	0.08 (0.02–0.30)	
Timepoint						
At diagnosis	3	95	< 0.001	38.82	0.26 (0.07–0.95)	
Pre‐treatment	21	98	< 0.001	17.83	0.32 (0.18–0.58)	

### 3.4 Meta‐Analysis of Prognostic Outcome in Patients With Gynaecological Tumour‐Associated Sarcopenia

A total of 11 studies [16, 17, 18, 19, 20, 21, 24, 25, 26, 27, 28, 30] were analysed for overall survival (OS) with a maximum follow‐up of 69.8 months, of which the results of 4 studies [16, 18, 19, 28] showed that OS in patients with gynaecologic tumours combined with sarcopenia was significantly lower than that in patients with gynaecologic tumours alone, 1 study [21] showed a significant difference in the 5‐year survival rate for OS (*p* = 0.001) and 2 studies [25, 30] showed no statistically significant difference. A study by Aust et al. [5] showed that age, international federation of gynaecology and obstetrics (FIGO) stage and suboptimal cytoreduction were independently associated with OS in patients with gynaecologic oncology combined with myopenia, of which 5 studies [17, 18, 24, 25, 30] analysed the progression‐free survival (PFS), which ranged from 3.5 to 26.5 months, and gynaecologic tumours alone had a PFS of 14.3–29.1 months. Three studies [17, 25, 30] showed no significant difference in the effect of whether gynaecologic tumours were combined with sarcopenia on PFS. Aust et al. [5] showed that the 5‐year PFS of gynaecologic tumours combined with sarcopenia was approximately 10.4% and that of gynaecologic tumours alone was 43.4%, with a significant difference (*p* < 0.0%). The difference was significant (*p* < 0.001). Four studies [19, 20, 26, S1] analysed the mortality rate of patients. They showed that the mortality rate of patients with gynaecologic tumours alone ranged from 1.5% to 33.9%. The mortality rate of patients with gynaecologic tumours combined with sarcopenia ranged from 2.9% to 36%, and all of them showed that the difference was not statistically significant. One study [20] analysed the length of hospital stay and showed that patients with gynaecologic tumours alone were hospitalized from 1.5% to 33.9%. The analysis of the hospital stay was 10–14.5 days for patients with gynaecologic tumours alone and 8–16.9 days for gynaecologic tumours combined with sarcopenia, and the two studies did not describe the difference in results.

### 3.5 Sensitivity Analysis and Publication bias Detection

The sensitivity analysis, conducted by sequentially excluding studies with *I* ^2^ > 50%, confirmed the stability of the results. After applying the random‐effects model, the prevalence of sarcopenia in gynaecologic oncology patients was 38.8% (*I* ^2^ = 96%, 95% CI [0.49–0.79], *p* < 0.001), consistent with the study's overall findings, indicating robustness in the meta‐analysis results. Funnel plot analysis revealed asymmetry in the distribution of study sites, and Egger's test, with a result of *p* < 0.001, suggested the presence of potential publication bias. The funnel plot is shown in Figure S6.

## 4 Discussion

Sarcopenia is more prevalent among patients with tumours. On one hand, tumours as consumptive diseases significantly compromise the patient's physical condition. On the other hand, the side effects of cancer treatments often place patients in a state of physical inactivity, contributing to muscle atrophy. Furthermore, specific treatments, such as chemotherapy, can directly damage muscle tissue, exacerbating the development of sarcopenia [3, S1].

The prevalence of sarcopenia among gynaecologic oncology patients was notably high across the 24 studies included in this analysis, ranging from approximately 9.5% to 62.7%, with a combined effect size of 38.8% (*I* ^2^ = 96%, 95% CI [0.49–0.79], *p* < 0.001). Sarcopenia is a crucial indicator of both nutritional status and physical functionality in these patients. Variations in sarcopenia prevalence across different countries may be due to differences in healthcare quality, economic development and racial factors. Additionally, the limited number of cross‐sectional studies on sarcopenia in gynaecologic cancers, both in China and internationally, may introduce some bias. The result of Egger's test (*p* < 0.001) indicates the potential presence of publication bias. Notably, removing studies that contributed to the asymmetry observed in the funnel plot did not alter the overall results. This bias may be attributed to factors such as the timing of sarcopenia diagnosis, variations in diagnostic criteria and the small sample sizes of the included studies.

The subgroup analysis of this study revealed a higher prevalence of sarcopenia among gynaecologic oncology patients aged over 60 years with a BMI greater than 25 kg/m^2^. This finding is consistent with previous research and may be attributed to several factors. As individuals age, both muscle mass and anabolic capacity decline, contributing to a higher prevalence of sarcopenia in this demographic. Liu et al. [S2] analysed the prevalence of obesity and sarcopenia in 1637 older adults from a community‐based outpatient clinic in Hong Kong. They suggested that obesity acted as a protective factor for sarcopenia when defined by BMI but as a risk factor when defined by the percentage of body fat. This indicates that using BMI alone to assess obesity in older adults may be inadequate, as BMI only measures body weight without considering body composition. Linge et al. [S3] recommended a more comprehensive understanding of optimal body composition to balance fat and lean body mass. The ‘obesity paradox’ further suggests that, despite the association between higher BMI and increased cancer risk, overweight (BMI 25–30 kg/m^2^) and Class 1 obese (BMI 30–35 kg/m^2^) patients may have a lower overall risk of mortality following a cancer diagnosis [S4].

Different diagnostic criteria for sarcopenia have led to different conclusions. According to the requirements promulgated by the 2018 EWGSOP criteria [2], the 2019 AWGS criteria [3] and the International Working Group on Sarcopenia (IWGS) [S5] as a reference to formulate clinical diagnostic guideline recommendations for sarcopenia, the diagnosis of sarcopenia requires the assessment of the patient's muscle strength, muscle mass and physical fitness level, which is comprehensively judged by combining the indicators of the above three aspects. Most of the literature included in this study used SMI to diagnose sarcopenia, but the diagnostic criteria were not standardized and were 38.5–41.5 cm^2^/m^2^. Zhuang et al. [S6] found that sarcopenia was a strong predictor of 5‐year survival in 937 patients with gastric cancer during long‐term follow‐up. They determined that the critical values for sarcopenia, measured by L3 SMI, were 40.8 cm^2^/m^2^ for men and 34.9 cm^2^/m^2^ for women. These criteria have been widely cited and applied in domestic and international studies [S7]. The thresholds for diagnosing sarcopenia vary across different diseases and racial groups. Future research involving high‐quality, large‐sample studies is needed to validate the reliability of these diagnostic thresholds across diverse populations.

An increasing number of studies have concluded that tumour‐associated sarcopenia not only interacts with antitumor therapies but also correlates with treatment toxicity and prognosis in cancer patients [S8–S10]. The results of this study indicated that OS was significantly lower in patients with gynaecologic tumours who also had sarcopenia compared to those with gynaecologic tumours alone. In an initial study by Huang et al. [S11] involving 394 cases of non‐metastatic nasopharyngeal carcinoma, it was found that severe skeletal muscle loss was associated with a reduced OS in patients (HR = 2.79, *p* = 0.002). Subsequently, Hua et al. [S8] conducted a retrospective analysis of 862 patients with nasopharyngeal carcinoma using L3 SMI calculated from CT images. The patients were categorized into a sarcopenia group (19.7%) and a non‐sarcopenia group (80.3%) based on a threshold of 18.82 cm^2^/m^2^, and these findings were validated in a matched cohort of 308 patients. The study found that the 5‐year OS and PFS were significantly worse in patients with sarcopenia compared to those without. Multivariate analysis revealed that sarcopenia was an independent prognostic factor for OS and distant metastasis‐free survival (DMFS). Additionally, the response rate to treatment was significantly lower in sarcopenic patients, and the prevalence of treatment‐related toxicities was notably higher. The mortality rate among patients in this study did not demonstrate a statistically significant difference. Previous research has indicated that patients with sarcopenia are at increased risk for decreased function, reduced mobility, frailty, falls, fractures and hospital readmissions, all of which can contribute to a higher risk of mortality. A recent meta‐analysis has shown that sarcopenia can independently increase the risk of death, regardless of population, diagnostic criteria or follow‐up duration [4]. However, the prognostic impact of sarcopenia in gynaecological oncology patients requires further validation in studies with larger sample sizes. In addition to these effects, sarcopenia also influences the length of hospital stays. A study by Kim et al. [S12] involving 121 patients infected with the novel coronavirus found that the hospitalization duration for patients with comorbid sarcopenia was significantly longer than for those without. In the present study, however, the hospitalization duration was not reported as statistically significant, which may be attributed to the limited number of studies that included relevant indices and the small sample sizes involved.

This study has several limitations: (1) The heterogeneity among the included studies was high, and it was not significantly reduced despite conducting subgroup analyses. (2) The number and sample size of studies in some subgroups were small, which may impact the reliability of the findings. (3) Grey literature was not included, leading to potential publication bias in the included studies; thus, the results need further validation. (4) The subgroup analysis only highlighted differences in the prevalence of sarcopenia among gynaecologic oncology patients, without addressing high‐risk factors or prognostic indicators. These aspects require further investigation through large‐scale cohort studies and high‐quality randomized controlled trials.

## 5 Conclusion

Meta‐analysis revealed a higher prevalence of sarcopenia among gynaecologic oncology patients. Current research on sarcopenia and its impact on tumour prognosis is still in the exploratory phase, with inconsistent evaluation methods across different types of tumours. Healthcare professionals should enhance screening for sarcopenia, identify high‐risk patients early and implement appropriate preventive measures. Although most studies recognize the influence of sarcopenia on tumour prognosis, many are retrospective and have limited capacity for establishing causality. Thus, further research is needed to confirm these findings and improve understanding.

## Ethics Statement

The authors have nothing to report.

## Consent

The authors have nothing to report.

## Conflicts of Interest

The authors declare no conflicts of interest.

## Supporting information


**Table S1.** Search formula using PubMed as an example.
**Table S2.** Literature quality assessment results.
**Figure S1.** Subgroup analysis of disease diagnosis.
**Figure S2.** Subgroup analysis of age.
**Figure S3.** Subgroup analysis of BMI.
**Figure S4.** Subgroup analysis of diagnostic criteria for sarcopenia.
**Figure S5.** Subgroup analysis of measurement positions.
**Figure S6.** Measure timepoint.
**Figure S7.** Funnel plot.

**Data S1.** Supporting Information.

## Data Availability

The authors have nothing to report.

## References

[1] A. Tong , K. Flemming , E. McInnes , S. Oliver , and J. Craig , “Enhancing Transparency in Reporting the Synthesis of Qualitative Research: ENTREQ,” BMC Medical Research Methodology 12 (2012): 181. 23185978 10.1186/1471-2288-12-181 PMC3552766

[2] H. J. Schünemann , A. D. Oxman , J. Brozek , et al., “Grading Quality of Evidence and Strength of Recommendations for Diagnostic Tests and Strategies,” BMJ 336 (2008): 1106–1110. 18483053 10.1136/bmj.39500.677199.AE PMC2386626

[3] A. Stang , “Critical Evaluation of the Newcastle‐Ottawa Scale for the Assessment of the Quality of Nonrandomized Studies in Meta‐Analyses,” European Journal of Epidemiology 25 (2010): 603–605. 20652370 10.1007/s10654-010-9491-z

[4] B. Ataseven , T. G. Luengo , A. du Bois , et al., “Skeletal Muscle Attenuation (Sarcopenia) Predicts Reduced Overall Survival in Patients With Advanced Epithelial Ovarian Cancer Undergoing Primary Debulking Surgery,” Annals of Surgical Oncology 25 (2018): 3372–3379. 30069659 10.1245/s10434-018-6683-3

[5] S. Aust , T. Knogler , D. Pils , et al., “Skeletal Muscle Depletion and Markers for Cancer Cachexia Are Strong Prognostic Factors in Epithelial Ovarian Cancer,” PLoS ONE 10 (2015): e0140403. 26457674 10.1371/journal.pone.0140403 PMC4601693

[6] H. Bronger , P. Hederich , A. Hapfelmeier , et al., “Sarcopenia in Advanced Serous Ovarian Cancer,” International Journal of Gynecological Cancer 27 (2017): 223–232. 27870708 10.1097/IGC.0000000000000867

[7] S. H. Chae , C. Lee , S. H. Yoon , et al., “Sarcopenia as a Predictor of Prognosis in Early Stage Ovarian Cancer,” Journal of Korean Medical Science 36 (2021): e2. 33398939 10.3346/jkms.2021.36.e2 PMC7781849

[8] L. B. Conrad , H. Awdeh , S. Acosta‐Torres , et al., “Pre‐Operative Core Muscle Index in Combination With Hypoalbuminemia Is Associated With Poor Prognosis in Advanced Ovarian Cancer,” Journal of Surgical Oncology 117 (2018): 1020–1028. 29409111 10.1002/jso.24990

[9] H. Donkers , K. E. Fasmer , J. McGrane , et al., “The Role of Sarcopenic Obesity in High‐Grade Endometrial Cancer,” International Journal of Gynaecology and Obstetrics 154 (2021): 248–255. 33445216 10.1002/ijgo.13591

[10] P. P. Fadadu , C. L. Polen‐De , M. E. McGree , et al., “Patients Triaged to Neoadjuvant Chemotherapy Have Higher Rates of Sarcopenia: An Opportunity for Prehabilitation,” Gynecologic Oncology 160 (2021): 40–44. 33109391 10.1016/j.ygyno.2020.10.025

[11] L. Flores‐Cisneros , L. Cetina‐Pérez , L. Castillo‐Martínez , et al., “Body Composition and Nutritional Status According to Clinical Stage in Patients With Locally Advanced Cervical Cancer,” European Journal of Clinical Nutrition 75 (2021): 852–855. 33149254 10.1038/s41430-020-00797-y

[12] C. Y. Huang , Y. C. Yang , T. C. Chen , et al., “Muscle Loss During Primary Debulking Surgery and Chemotherapy Predicts Poor Survival in Advanced‐Stage Ovarian Cancer,” Journal of Cachexia, Sarcopenia and Muscle 11 (2020): 534–546. 31999069 10.1002/jcsm.12524 PMC7113537

[13] A. Kumar , M. R. Moynagh , F. Multinu , et al., “Muscle Composition Measured by CT Scan Is a Measurable Predictor of Overall Survival in Advanced Ovarian cancer,” Gynecologic Oncology 142 (2016): 311–316. 27235857 10.1016/j.ygyno.2016.05.027

[14] L. M. Kuroki , M. Mangano , J. E. Allsworth , et al., “Pre‐Operative Assessment of Muscle Mass to Predict Surgical Complications and Prognosis in Patients With Endometrial Cancer,” Annals of Surgical Oncology 22 (2015): 972–979. 25190123 10.1245/s10434-014-4040-8 PMC4355998

[15] J. Nattenmüller , J. Rom , T. Buckner , et al., “Visceral Abdominal fat Measured by Computer Tomography as a Prognostic Factor for Gynecological Malignancies?,” Oncotarget 9 (2018): 16330–16342. 29662648 10.18632/oncotarget.24667 PMC5893243

[16] I. J. Rutten , D. P. van Dijk , R. F. Kruitwagen , R. G. Beets‐Tan , S. W. Olde Damink , and T. van Gorp , “Loss of Skeletal Muscle During Neoadjuvant Chemotherapy Is Related to Decreased Survival in Ovarian Cancer Patients,” Journal of Cachexia, Sarcopenia and Muscle 7 (2016): 458–466. 27030813 10.1002/jcsm.12107 PMC4782251

[17] M. Sánchez , D. Castro‐Eguiluz , J. Luvián‐Morales , et al., “Deterioration of Nutritional Status of Patients With Locally Advanced Cervical Cancer During Treatment With Concomitant Chemoradiotherapy,” Journal of Human Nutrition and Dietetics 32 (2019): 480–491. 30938007 10.1111/jhn.12649

[18] S. A. Staley , K. Tucker , M. Newton , et al., “Sarcopenia as a Predictor of Survival and Chemotoxicity in Patients With Epithelial Ovarian Cancer Receiving Platinum and Taxane‐Based Chemotherapy,” Gynecologic Oncology 156 (2020): 695–700. 31928805 10.1016/j.ygyno.2020.01.003

[19] J. Ubachs , S. N. Koole , M. Lahaye , et al., “No Influence of Sarcopenia on Survival of Ovarian Cancer Patients in a Prospective Validation Study,” Gynecologic Oncology 159 (2020): 706–711. 33019981 10.1016/j.ygyno.2020.09.042

[20] J. Ubachs , W. van de Worp , R. D. W. Vaes , et al., “Ovarian Cancer Ascites Induces Skeletal Muscle Wasting In Vitro and Reflects Sarcopenia in Patients,” Journal of Cachexia, Sarcopenia and Muscle 13 (2022): 311–324. 34951138 10.1002/jcsm.12885 PMC8818657

[21] R. Yamada , Y. Todo , H. Kurosu , et al., “Validity of Measuring Psoas Muscle Mass Index for Assessing Sarcopenia in Patients With Gynecological Cancer,” Japanese Journal of Clinical Oncology 51 (2021): 393–399. 33306784 10.1093/jjco/hyaa218

[22] R. Yamada , Y. Todo , K. Minowa , et al., “Prevalence of Sarcopenia in Patients With Gynecological Cancer,” Japanese Journal of Clinical Oncology 52 (2022): 1001–1007. 35661218 10.1093/jjco/hyac087

[23] N. Yoshikawa , A. Shirakawa , K. Yoshida , et al., “Sarcopenia as a Predictor of Survival Among Patients With Organ Metastatic Cervical Cancer,” Nutrition in Clinical Practice 35 (2020): 1041–1046. 32253779 10.1002/ncp.10482

[24] T. Yoshikawa , M. Takano , M. Miyamoto , et al., “Psoas Muscle Volume as a Predictor of Peripheral Neurotoxicity Induced by Primary Chemotherapy in Ovarian Cancers,” Cancer Chemotherapy and Pharmacology 80 (2017): 555–561. 28726081 10.1007/s00280-017-3395-5

[25] Y. Yoshino , A. Taguchi , Y. Nakajima , et al., “Extreme Skeletal Muscle Loss During Induction Chemotherapy Is an Independent Predictor of Poor Survival in Advanced Epithelial Ovarian Cancer Patients,” Journal of Obstetrics and Gynaecology Research 46 (2020): 2662–2671. 33015913 10.1111/jog.14516

[26] V. van der Zanden , N. J. van Soolingen , A. R. Viddeleer , et al., “Low Preoperative Skeletal Muscle Density Is Predictive for Negative Postoperative Outcomes in Older Women With Ovarian Cancer,” Gynecologic Oncology 162 (2021): 360–367. 34112514 10.1016/j.ygyno.2021.05.039

[27] N. Silva de Paula , B. K. de Aguiar , M. Azevedo Aredes , and G. Villaça Chaves , “Sarcopenia and Skeletal Muscle Quality as Predictors of Postoperative Complication and Early Mortality in Gynecologic Cancer,” International Journal of Gynecological Cancer 28 (2018): 412–420. 29266018 10.1097/IGC.0000000000001157

[28] G. R. Williams , R. F. Dunne , S. Giri , S. S. Shachar , and B. J. Caan , “Sarcopenia in the Older Adult With Cancer,” Journal of Clinical Oncology 39 (2021): 2068–2078. 34043430 10.1200/JCO.21.00102 PMC8260902

[29] J. Linge , S. B. Heymsfield , and L. O. Dahlqvist , “On the Definition of Sarcopenia in the Presence of Aging and Obesity‐Initial Results From UK Biobank,” Journals of Gerontology. Series A, Biological Sciences and Medical Sciences 75 (2020): 1309–1316. 31642894 10.1093/gerona/glz229 PMC7302181

[30] C. Liu , D. Dhindsa , Z. Almuwaqqat , Y. V. Sun , and A. A. Quyyumi , “Very High High‐Density Lipoprotein Cholesterol Levels and Cardiovascular Mortality,” American Journal of Cardiology 167 (2022): 43–53. 35039162 10.1016/j.amjcard.2021.11.041

[31] B. J. Caan , E. M. Cespedes Feliciano , and C. H. Kroenke , “The Importance of Body Composition in Explaining the Overweight Paradox in Cancer‐Counterpoint,” Cancer Research 78 (2018): 1906–1912. 29654153 10.1158/0008-5472.CAN-17-3287 PMC5901895

[32] E. Dent , J. E. Morley , A. J. Cruz‐Jentoft , et al., “International Clinical Practice Guidelines for Sarcopenia (ICFSR): Screening, Diagnosis and Management,” Journal of Nutrition, Health & Aging 22 (2018): 1148–1161.10.1007/s12603-018-1139-930498820

[33] C. L. Zhuang , D. D. Huang , W. Y. Pang , et al., “Sarcopenia Is an Independent Predictor of Severe Postoperative Complications and Long‐Term Survival After Radical Gastrectomy for Gastric Cancer: Analysis From a Large‐Scale Cohort,” Medicine (Baltimore) 95 (2016): e3164. 27043677 10.1097/MD.0000000000003164 PMC4998538

[34] Z. Yang , X. Zhou , B. Ma , Y. Xing , X. Jiang , and Z. Wang , “Predictive Value of Preoperative Sarcopenia in Patients With Gastric Cancer: A Meta‐Analysis and Systematic Review,” Journal of Gastrointestinal Surgery 22 (2018): 1890–1902. 29987739 10.1007/s11605-018-3856-0

[35] X. Hua , J. F. Liao , X. Huang , et al., “Sarcopenia Is Associated With Higher Toxicity and Poor Prognosis of Nasopharyngeal Carcinoma,” Therapeutic Advances in Medical Oncology 12 (2020): 1758835920947612. 32913446 10.1177/1758835920947612 PMC7444117

[36] F. Bozzetti , “Chemotherapy‐Induced Sarcopenia,” Current Treatment Options in Oncology 21 (2020): 7. 32002684 10.1007/s11864-019-0691-9

[37] M. C. Vega , A. Laviano , and G. D. Pimentel , “Sarcopenia and Chemotherapy‐Mediated Toxicity,” einstein (Sao Paulo) 14 (2016): 580–584. 28076611 10.1590/S1679-45082016MD3740 PMC5221390

[38] X. Huang , J. Ma , L. Li , and X. D. Zhu , “Severe Muscle Loss During Radical Chemoradiotherapy for Non‐metastatic Nasopharyngeal Carcinoma Predicts Poor Survival,” Cancer Medicine 8 (2019): 6604–6613. 31517443 10.1002/cam4.2538 PMC6825977

[39] M. C. Kim , Y. Lim , S. H. Lee , et al., “Early Left Ventricular Unloading or Conventional Approach After Venoarterial Extracorporeal Membrane Oxygenation: The EARLY‐UNLOAD Randomized Clinical Trial,” Circulation 148 (2023): 1570–1581. 37850383 10.1161/CIRCULATIONAHA.123.066179

