# Supplementary material for: Prevalence and Prognostic Significance of Sarcopenia in Gynecologic Oncology: A Systematic Review and Meta‐Analysis
Source: J Cachexia Sarcopenia Muscle. 2025 Feb 2;16(1):e13699. doi: 10.1002/jcsm.13699 (PMC11788493; doi:10.1002/jcsm.13699)
Supplement: Supplementary file 1 — Table S1. Search formula using PubMed as an example. Table S2. Literature quality assessment results. Figure S1. Subgroup analysis of disease diagnosis. Figure S2. Subgroup analysis of age. Figure S3. Subgroup analysis of BMI. Figure S4. Subgroup analysis of diagnostic criteria for sarcopenia. Figure S5. Subgroup analysis of measurement positions. Figure S6. Measure timepoint. Figure S7. Funnel plot. [file JCSM-16-e13699-s001.docx]

Table1 Search formula using PubMed as an example.

1. "sarcopenia"[MeSH Terms]
2. "myopenia"[Title/Abstract] OR "pachyonychia"[Title/Abstract] OR "nutrition status"[Title/Abstract] OR "human body"[MeSH Terms] OR "compostion"[All Fields] OR "measures"[Title/Abstract] OR "body weight"[Title/Abstract] OR "body composition*"[Title/Abstract] OR "skeletal muscle depletion"[Title/Abstract] OR "skeletal muscle quality"[Title/Abstract] OR "skeletal muscle attenuation"[Title/Abstract] OR "skeletal muscle*"[Title/Abstract] OR "core"[All Fields] OR "muscle"[Title/Abstract] OR "BIA"[Title/Abstract] OR "phase angle"[Title/Abstract]
3. #1 OR #2
4. "overian cancer"[Mesh] OR "endometrial neoplasms"[Mesh] OR "uterine neoplasms"[Mesh] OR "uterine cervical neoplasms"[Mesh] OR "genital neoplasms, female"[Mesh] OR "vaginal neoplasms"[Mesh] OR "vulvar neoplasms"[Mesh] OR "choriocarcinoma, non-gestational"[Mesh] OR "choriocarcinoma"[Mesh]
5. endometrial neoplasms*[Title/Abstract] or uterine neoplasms*[Title/Abstract] or vaginal neoplasms*[Title/Abstract] OR gynecologic oncology [Title/Abstract]
6. #4 OR #5

#3 AND #6

Table2 Literature quality assessment results

| Study | Selection of Research Subjects | | | | | Outcome Measurement | | | Totals |
| --- | --- | --- | --- | --- | --- | --- | --- | --- | --- |
|  | Representativeness of the exposed group | Representation of non-exposed groups | Exposure factor determination | Outcome indicators not yet available for observation at the time of the study | Between-group comparability (does it control for confounders） | Blind independent evaluation | Sufficiently long follow-up | Follow-up completeness |  |
| Aust et al. (2015) | 1 | 1 | 1 | 0 | 2 | 1 | 1 | 1 | 8 |
| Kuroki et al. (2015) | 1 | 1 | 1 | 0 | 2 | 0 | 1 | 1 | 7 |
| Kumar et al. (2016) | 1 | 1 | 1 | 0 | 2 | 0 | 1 | 1 | 7 |
| Bronger et al.(2016) | 1 | 1 | 1 | 0 | 2 | 0 | 1 | 1 | 7 |
| Rutten et al. (2016) | 1 | 1 | 1 | 0 | 2 | 1 | 1 | 1 | 8 |
| Silva de Paula et al.(2017) | 1 | 1 | 1 | 0 | 2 | 0 | 1 | 1 | 7 |
| Conrad et al. (2017) | 1 | 1 | 1 | 0 | 2 | 1 | 1 | 1 | 8 |
| Yoshikawa et al. (2017) | 1 | 1 | 1 | 1 | 2 | 0 | 0 | 1 | 7 |
| Ataseven et al. (2018) | 1 | 1 | 1 | 0 | 2 | 1 | 0 | 1 | 7 |
| Nattenmüller et al. (2018) | 1 | 1 | 1 | 0 | 2 | 0 | 0 | 1 | 6 |
| Sánchez et al. (2019) | 1 | 1 | 1 | 1 | 2 | 0 | 1 | 1 | 8 |
| Yamada et al.(2022) | 1 | 1 | 1 | 1 | 2 | 0 | 0 | 1 | 7 |
| Huang et al. (2020) | 1 | 1 | 1 | 0 | 1 | 0 | 1 | 1 | 6 |
| Fadadu et al. (2020) | 1 | 1 | 1 | 1 | 2 | 0 | 0 | 1 | 7 |
| Donkers et al.(2020) | 1 | 1 | 1 | 0 | 2 | 0 | 1 | 1 | 7 |
| Staley et al.(2020) | 1 | 1 | 1 | 0 | 2 | 1 | 1 | 1 | 8 |
| Yoshikawa et al. (2020) | 1 | 1 | 1 | 0 | 2 | 1 | 0 | 1 | 7 |
| Flores-Cisneros et al. (2020) | 1 | 1 | 1 | 1 | 2 | 0 | 0 | 1 | 7 |
| Yoshino et al. (2020) | 1 | 1 | 1 | 0 | 2 | 1 | 1 | 1 | 8 |
| Ubachs et al.(2020) | 1 | 1 | 1 | 0 | 2 | 1 | 0 | 1 | 7 |
| Chae et al. (2021) | 1 | 1 | 1 | 0 | 2 | 0 | 1 | 1 | 7 |
| Van der Zanden et al. (2021) | 1 | 1 | 1 | 0 | 2 | 1 | 1 | 1 | 8 |
| Ubachs et al. (2022) | 1 | 1 | 1 | 1 | 2 | 0 | 1 | 1 | 8 |

Subgroup analysis affecting the prevalence of patients with gynecologic tumors combined with sarcopenia


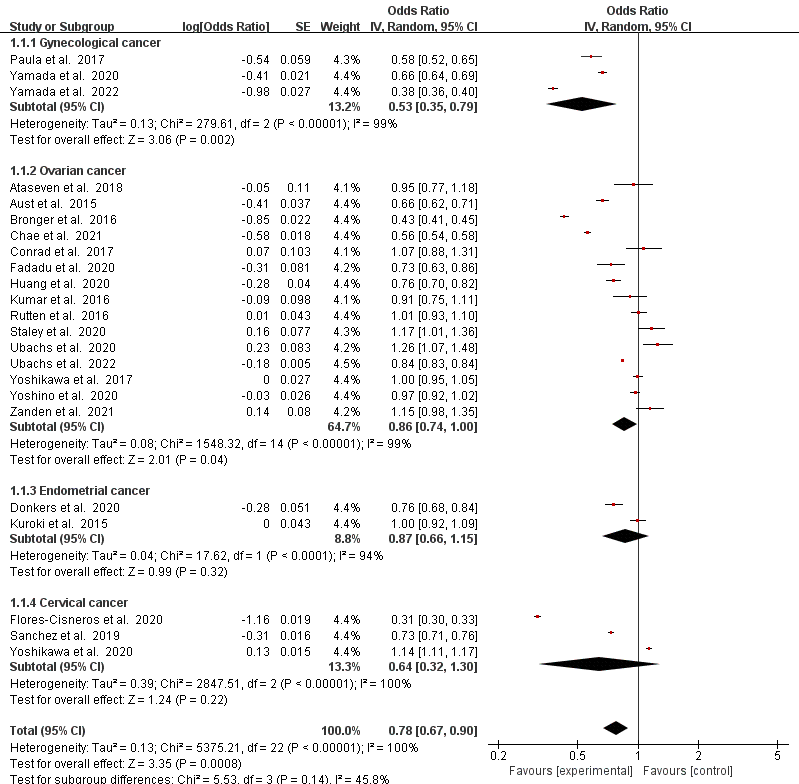


**Figure1 Subgroup analysis of disease diagnosis**


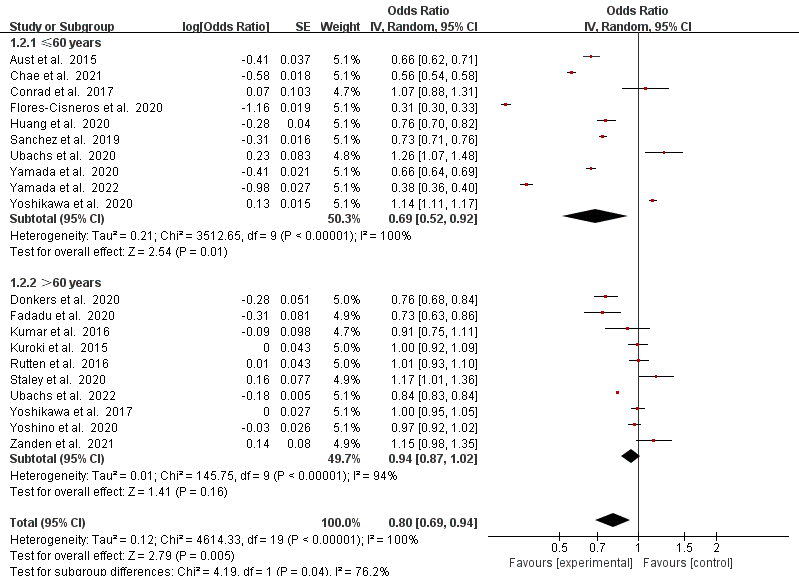


**Figure2 Subgroup analysis of age**


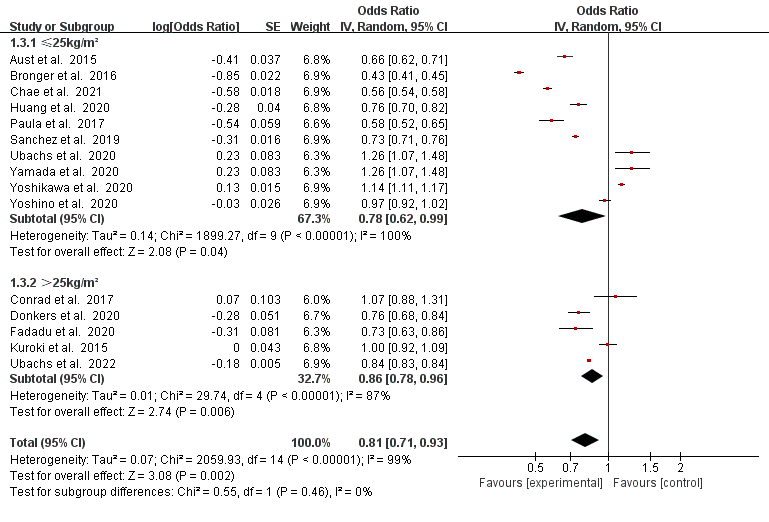


**Figure3 Subgroup analysis of BMI**


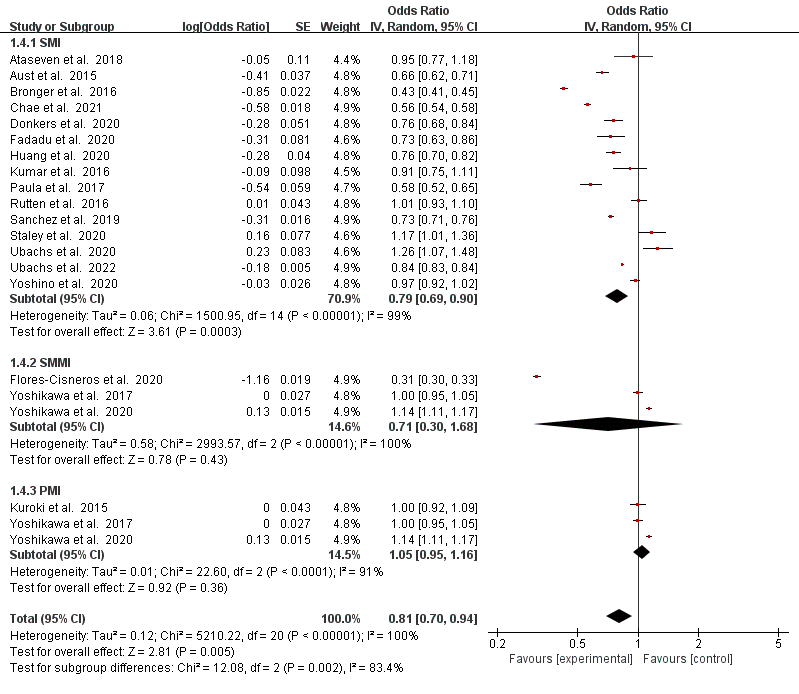


**Figure4 Subgroup analysis of diagnostic criteria for sarcopenia**


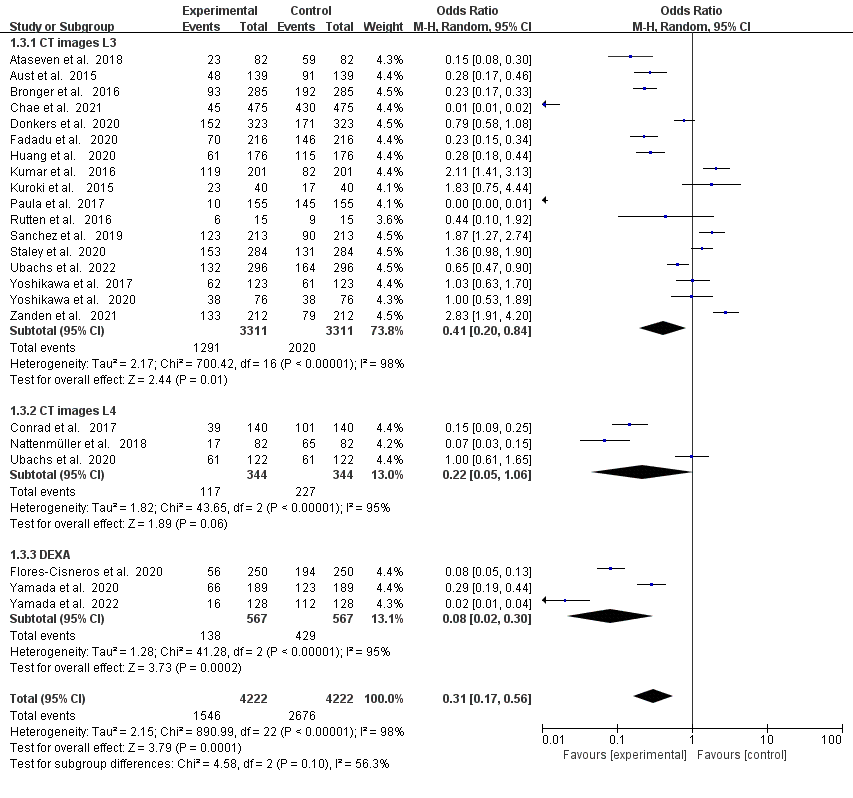


**Figure5 Subgroup analysis of measurement positions**


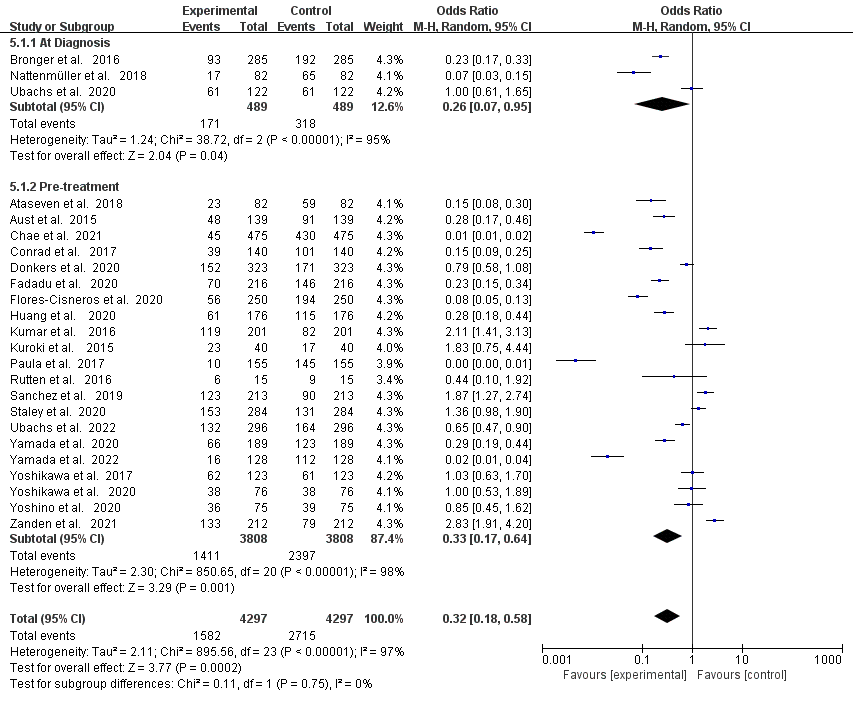


**Figure 6 Measure timepoint**


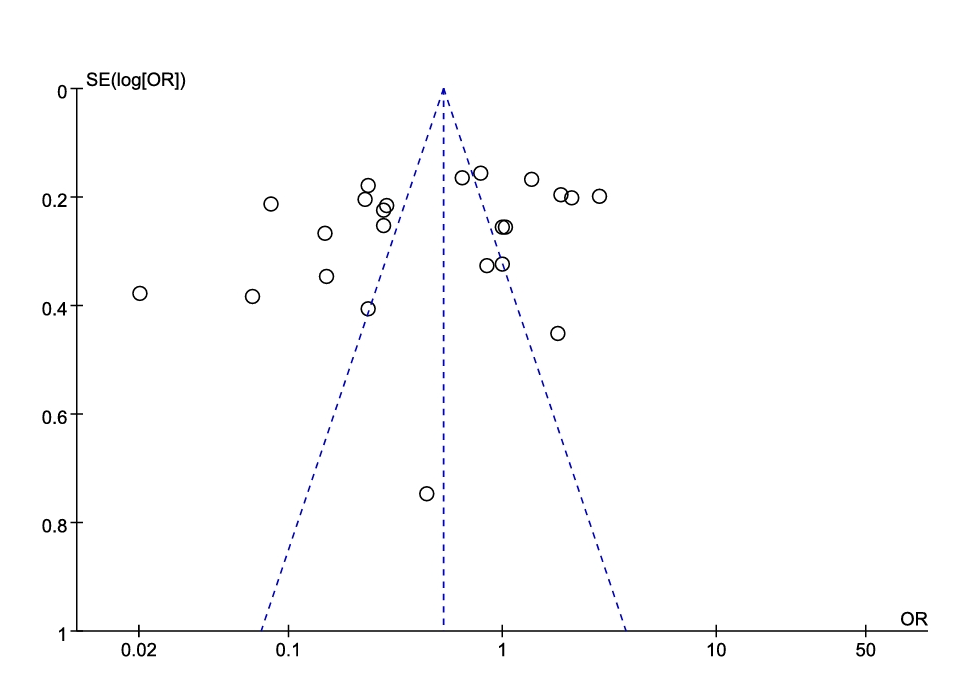


Figure 7 Funnel plot
