# Supplementary material for: Prevalence and Prognostic Significance of Sarcopenia in Gynecologic Oncology: A Systematic Review and Meta‐Analysis
Source: J Cachexia Sarcopenia Muscle. 2025 Feb 2;16(1):e13699. doi: 10.1002/jcsm.13699 (PMC11788493; doi:10.1002/jcsm.13699)
Supplement: Supplementary file 2 — Data S1. Supporting Information. [file JCSM-16-e13699-s002.docx]

**Additional references**

S1. Arai H. Sarcopenia diagnostic criteria update by EWGSOP: what has been changed? Eur Geriatr Med. 2018;9:733-4.

S2. Liu C, Dhindsa D, Almuwaqqat Z, Sun YV, Quyyumi AA. Very High High-Density Lipoprotein Cholesterol Levels and Cardiovascular Mortality. Am J Cardiol. 2022;167:43-53.

S3. Linge J, Heymsfield SB, Dahlqvist Leinhard O. On the Definition of Sarcopenia in the Presence of Aging and Obesity-Initial Results from UK Biobank. J Gerontol A Biol Sci Med Sci. 2020;75:1309-1316.

S4. Caan BJ, Cespedes Feliciano EM, Kroenke CH. The Importance of Body Composition in Explaining the Overweight Paradox in Cancer-Counterpoint. Cancer Res. 2018;78:1906-1912.

S5. Dent E, Morley JE, Cruz-Jentoft AJ, Arai H, Kritchevsky SB, Guralnik J, et al. International Clinical Practice Guidelines for Sarcopenia (ICFSR): Screening, Diagnosis and Management. J Nutr Health Aging. 2018;22:1148-1161.

S6. Zhuang CL, Huang DD, Pang WY, Zhou CJ, Wang SL, Lou N, et al. Sarcopenia is an Independent Predictor of Severe Postoperative Complications and Long-Term Survival After Radical Gastrectomy for Gastric Cancer: Analysis from a Large-Scale Cohort. Medicine (Baltimore). 2016;95:e3164.

S7. Yang Z, Zhou X, Ma B, Xing Y, Jiang X, Wang Z. Predictive Value of Preoperative Sarcopenia in Patients with Gastric Cancer: a Meta-analysis and Systematic Review. J Gastrointest Surg. 2018;22:1890-1902.

S8. Hua X, Liao JF, Huang X, Huang HY, Wen W, Long ZQ, et al. Sarcopenia is associated with higher toxicity and poor prognosis of nasopharyngeal carcinoma. Ther Adv Med Oncol. 2020;12:1758835920947612.

S9. Bozzetti F. Chemotherapy-Induced Sarcopenia. Curr Treat Options Oncol. 2020;21:7.

S10. Vega MC, Laviano A, Pimentel GD. Sarcopenia and chemotherapy-mediated toxicity. Einstein (Sao Paulo). 2016;14:580-584.

S11. Huang X, Ma J, Li L, Zhu XD. Severe muscle loss during radical chemoradiotherapy for non-metastatic nasopharyngeal carcinoma predicts poor survival. Cancer Med. 2019;8:6604-6613.

S12. Kim MC, Lim Y, Lee SH, Shin Y, Ahn JH, Hyun DY, et al. Early Left Ventricular Unloading or Conventional Approach After Venoarterial Extracorporeal Membrane Oxygenation: The EARLY-UNLOAD Randomized Clinical Trial. Circulation. 2023;148:1570-1581.
